# Gut microbiome components predict response to neoadjuvant short-course radiotherapy followed by camrelizumab and chemotherapy in locally advanced rectal cancer (UNION): a prospective study

**DOI:** 10.3389/fphar.2026.1829108

**Published:** 2026-05-29

**Authors:** Qiai You, Min Jin, Bin Zhou, Chuying Huang, Zhenyu Lin, Jianli Hu, Jun Xue, Xinyi Chen, Yilin Xiao, Ruihan Li, Yan Zong, Mengjiao Wu, Tao Zhang, Hongli Liu

**Affiliations:** 1 Cancer Center, Union Hospital, Tongji Medical College, Huazhong University of Science and Technology, Wuhan, China; 2 Hubei Key Laboratory of Precision Radiation Oncology, Wuhan, China; 3 Institute of Radiation Oncology, Union Hospital, Tongji Medical College, Huazhong University of Science and Technology, Wuhan, China; 4 Hubei Key Laboratory for Translational Research in Traditional Chinese Medicine, The Central Hospital of Enshi Tujia and Miao Autonomous Prefecture, Hubei Minzu University, Enshi, China; 5 School of Public Health, Xiamen University, Xiamen, Fujian, China

**Keywords:** biomarker, camrelizumab, gut microbiome, locally advanced rectal cancer, neoadjuvant therapy

## Abstract

**Background:**

Although the gut microbiome shapes responses to anti-tumor immunotherapy and chemotherapy, its predictive value for neoadjuvant short-course radiotherapy (SCRT) followed by camrelizumab (CAM) and CAPOX in patients with locally advanced rectal cancer (LARC) has not been defined. This exploratory study aimed to evaluate whether the gut microbiome is associated with response to neoadjuvant SCRT followed by CAM and CAPOX.

**Methods:**

We obtained a total of 77 fecal samples from 36 patients with LARC, including 17 assigned to the long-course chemoradiotherapy (LCRT) group and 19 to the SCRT group. Samples were collected at three time points: baseline, after radiotherapy, and after chemoimmunotherapy. DNA was extracted, followed by metagenomic sequencing to profile microbiota dynamics during neoadjuvant treatment.

**Results:**

In this pilot analysis, we observed significant differences in the gut microbiota between the SCRT and LCRT treatment cohorts. Specifically, *Bifidobacterium* and *Dorea* were significantly enriched following completion of SCRT sequential CAM and CAPOX therapy. Further analysis revealed that the relative abundances of these two genera changed significantly only before and after the SCRT regimen, with no notable changes observed in the LCRT group. Preliminary ROC analysis suggested potential utility of these taxa for predicting treatment response, though validation in larger cohorts is needed.

**Conclusion:**

The gut microbiome offers potential biomarkers that may stratify response to SCRT followed by CAM and CAPOX, representing a promising exploratory finding with potential clinical relevance.

**Clinical Trial Registration:**

https://clinicaltrials.gov/, identifier NCT04928807.

## 1 Introduction

Colorectal cancer (CRC) is the third most commonly diagnosed malignant tumor worldwide and the second leading cause of cancer-related mortality (Siegel et al., 2025), and many patients present with advanced-stage disease (Siegel et al., 2023). For patients with locally advanced rectal cancer (LARC), the current standard neoadjuvant treatment regimens include a comprehensive therapeutic approach combining short-course radiotherapy (SCRT) or long-course chemoradiotherapy (LCRT) with systemic chemotherapy. Nevertheless, significant scope persists for further enhancement of long-term oncological efficacy.

In our single-arm phase II trial, patients with LARC received preoperative SCRT followed by chemotherapy (CAPOX) and camrelizumab (CAM, a programmed cell death protein 1 monoclonal antibody), showing a pathological complete response (pCR) rate of 48.1% with an acceptable safety profile and favorable tolerability (Lin et al., 2021). Motivated by these promising outcomes, we initiated the UNION study, a phase III, open-label, multicenter randomized trial, aimed at evaluating the efficacy and safety of SCRT combined with CAM and CAPOX versus LCRT combined with CAPOX as neoadjuvant therapy for LARC. The early outcomes of this study revealed a significantly higher pCR rate in the SCRT arm (41.6% vs. 18.6%), accompanied by a favorable safety profile (Lin et al., 2024).

Accumulating evidence indicates that the gut microbiome and its metabolites are closely related to the formation, treatment and prognosis of colorectal cancer (Song et al., 2020). A prospective study found that the significant gut microbiome alterations between LARC patients who achieved pCR after neoadjuvant chemoradiotherapy and those who did not (Yang et al., 2024). More importantly, the gut microbiome may directly influence the efficacy of immunotherapy. Multimodal analysis of pan-cancer suggested that specific metabolites derived from gut microbiota can influence the systemic immune responses, thereby enhancing the efficacy of immunotherapy (Zhu et al., 2025).

Growing evidence suggests that the gut microbiome can reshape the tumor microenvironment (TME) and profoundly modulate antitumor immunity. *Bifidobacterium* has emerged as one of the most extensively studied bacteria associated with CRC pathogenesis. *B. adolescentis* activates Toll-like receptor 2 (TLR2) signaling to induce Decorin + macrophages that suppress CRC progression (Lin et al., 2023). Commensal bacteria of the *Lachnospiraceae* family, including *Ruminococcus gnavus* (*Rg*), *Blautia producta* (*Bp*), and *Dorea formicigenerans* (*Df*), are selectively enriched in normal colonic tissues, and tissue-resident *Rg* and *Bp* markedly inhibit the growth of colon cancer in immunocompetent mice, exerting antitumor effects by activating CD8^+^ T cells and maintaining their immune-surveillance function (Zhang et al., 2023). *Fusobacterium nucleatum* has been shown to drive M2 macrophage polarization within the TME established an immunosuppressive niche that promoted tumor immune evasion in colorectal cancer (Xu et al., 2021). It is also worth noting that immunotherapy can alter microbial community composition, leading to selective enrichment of specific bacterial species (Frankel et al., 2017). Collectively, dynamic alterations in the gut microbiome are closely linked to immunotherapy outcomes across diverse cancer types. Although gut microbiome signatures can predict response to neoadjuvant chemoradiotherapy (nCRT) in rectal cancer, their predictive value for neoadjuvant immunotherapy (NIT) remains unknown. Identifying microbial biomarkers to stratify patients and predict likely responders is urgently needed to enable precision treatment.

In this prospective study, we collected fecal samples from patients undergoing either SCRT chemoradiotherapy combined with immunotherapy or LCRT chemoradiotherapy. Metagenomic sequencing was employed to profile the gut microbiome and its functional gene expression at baseline, post-radiotherapy, and upon completion of neoadjuvant treatment. By integrating multi-database annotations including the Kyoto Encyclopedia of Genes and Genomes (KEGG) and carbohydrate-active enZymes (CAZy), we explored differential microbial species and their key functional pathways as potential predictive signatures. Preliminary machine-learning models were constructed to identify candidate microbial biomarkers for potential future development of precision screening approaches for SCRT followed by CAM and CAPOX, aiming to inform strategies to optimize therapeutic efficacy and minimize adverse events.

## 2 Methods

### 2.1 Participants and sample collection

A total of 77 fecal samples were collected from 36 Chinese patients enrolled in the UNION study, comprising 17 patients in the LCRT arm and 19 in the SCRT arm. The inclusion criteria were as follows: (1) aged 18–70 years; (2) newly diagnosed locally advanced rectal cancer; (3) no evidence of distant metastasis on imaging; (4) scheduled to receive neoadjuvant therapy for rectal cancer; (5) ability to tolerate chemoradiotherapy, immunotherapy, and surgical intervention; (6) no exposure to systemic antibiotics, immunosuppressants, corticosteroids and other drugs (proton pump inhibitors (PPIs), corticosteroids) affecting the gut microbiota within 4 weeks prior to baseline stool collection. The exclusion criteria were as follows: (1) concurrent history of other malignancies; (2) having other metabolic diseases (e.g., diabetes). Standardized medication protocol during treatment: during the treatment period, all patients received standardized antiemetic prophylaxis consisting of a single intravenous dose of dexamethasone (5 mg) prior to each chemotherapy cycle. This was a uniform intervention across both treatment arms. No other systemic antibiotics, immunosuppressants, or corticosteroids were administered during the study period except for this standardized premedication. This study was approved by the Ethics Committee of Tongji Medical College of Huazhong University of Science and Technology (approval No. 2021-0793), and all participants provided written informed consent prior to enrolment.

### 2.2 Fecal sample collection and DNA extraction

Fecal samples were collected before treatment, 3 days after radiotherapy, and 3 days after CAM plus CAPOX therapy or CAPOX alone. All samples were obtained from each participant within 30 min of defecation and immediately stored at −80 °C. Genomic DNA was extracted from fecal samples using the HiPure Stool DNA Mini Kit (Magen, Shanghai) according to the manufacturer’s protocol. Qubit fluorometer (Thermo Fisher Scientific, USA) was utilized for DNA concentration, and the integrity of the extracted genomic DNA was assessed via 1.5% agarose gel electrophoresis. The extracted DNA was required to have a concentration ≥20 ng/μL, a volume ≥20 μL, a total yield ≥400 ng, and exhibit intact or only mildly degraded integrity.

### 2.3 Metagenomic sequencing

0.5 μg qualified genomic DNA was randomly fragmented using a Bioruptor Pico, followed by size selection with magnetic beads. The DNA was repaired and adaptor was performed, followed by a second magnetic-bead purification step. The products were amplified and enriched by Polymerase Chain Reaction (PCR), and quality control was conducted prior to library construction. The double-stranded PCR libraries were purified, denatured, and circularized to generate single-stranded circular DNA (ssCirDNA). The DNA nanoballs (DNBs) were produced by rolling circle amplification (RCA) and then loaded onto chips using an automated system for immobilization. Sequencing was carried out on the DNBSEQ-T7 platform at Bioyi Biotechnology (Wuhan, China). Host (human) reads were removed by alignment to the human reference genome GCF_009914755.1 using Bowtie2 (v2.4.5) with the--very-sensitive preset.

### 2.4 Microbial community diversity analysis

Taxonomic abundances were normalized to relative abundance for cross-sample comparison. For α- and β-diversity analyses, no abundance filtering was applied to retain the complete community structure. For differential abundance analysis between groups, taxa with mean relative abundance <0.1% across samples were excluded to reduce noise and focus on biologically meaningful differences. Both α- and β-diversity metrics were applied to characterize microbial community variation. α-diversity, reflecting within-sample richness and evenness, was quantified using the Simpson and Chao1 indices. β-diversity, describing compositional differences between samples, was visualized and quantified by principal coordinate analysis (PCoA), and non-metric multidimensional scaling (NMDS), using Bray–Curtis distance. Additionally, principal component analysis (PCA) was performed based on Euclidean distance for comparison. To assess statistically significant differences in β diversity between groups, analysis of similarity (ANOSIM) was used. All calculations were performed in R.

### 2.5 Random-forest classification

Random-forest classification was performed using the randomForest package in R with the number of trees set to 500 (ntree = 500). The model performance was evaluated using 10-fold cross-validation, with an out-of-bag (OOB) error rate of 25%. Variable importance was assessed using mean decrease in Gini. The model was trained on metagenomic taxonomic profiles. This analysis was exploratory and the model was not externally validated in an independent cohort.

### 2.6 Functional annotation of non-redundant genes

After quality control, high-quality reads were *de novo* assembled with megahit or spades, the resulting contigs were taxonomically assigned by kraken2 (v2.1.2) against the NCBI NT (Nucleotide) database (build date: June 2023) and filtered to retain microbial genomes, MetaGeneMark predicted genes, CD-hit produced non-redundant gene and protein catalogs, and functional annotation was completed by Interproscan (TIGRFAM, Pfam, GO) and DIAMOND searches (KEGG, NR, Swiss-Prot, COG) with ≥30% alignment coverage (Buchfink et al., 2015; Aramaki et al., 2020). Genome-encoded proteins were extracted and aligned to the CAZy database with BLASTp. The CAZy database systematically classifies carbohydrate-active enzymes into six functional categories: glycoside hydrolases (GHs), glycosyl transferases (GTs), polysaccharide lyases (PLs), carbohydrate esterases (CEs), auxiliary activities (AAs), and carbohydrate-binding modules (CBMs) (Drula et al., 2022). Only the best hit exhibiting an alignment coverage of ≥30% was selected for the final annotation (Lombard et al., 2014). Leveraging KEGG and CAZy annotations together with non-redundant gene-abundance data, we compiled pathway-level gene tables for each sample in Python, and then employed LEfSe (LDA score > 2) to identify differentially enriched metabolic pathways.

### 2.7 ROC curve analysis

ROC curves were constructed to evaluate the predictive performance of selected microbial features for pCR status. The randomForest package in R was used to train random forest models on species relative abundance data. Sixty percent of the data were used for model training, and the remaining 40% were used for prediction and validation. The pROC package was then used to plot ROC curves and calculate AUC values.

### 2.8 Statistical analysis

For demographic and clinicopathological characteristics, categorical variables were compared using Pearson’s chi-square test or Fisher’s exact test, while continuous variables were analyzed using Student’s t-test. The Wilcoxon signed-rank test was used to calculate the significance for the metagenomic data. *p* values were adjusted for multiple comparisons using the Benjamini-Hochberg false discovery rate (FDR) procedure across taxa and functional pathways (q < 0.05 was considered significant); unadjusted *p* < 0.05 was used for all other comparisons. All statistical analyses were conducted using GraphPad Prism, version 8 (GraphPad Software, La Jolla, CA, USA) or R (v4.4.0).

## 3 Results

### 3.1 Clinical characteristics of LARC patients

**FIGURE 1:**
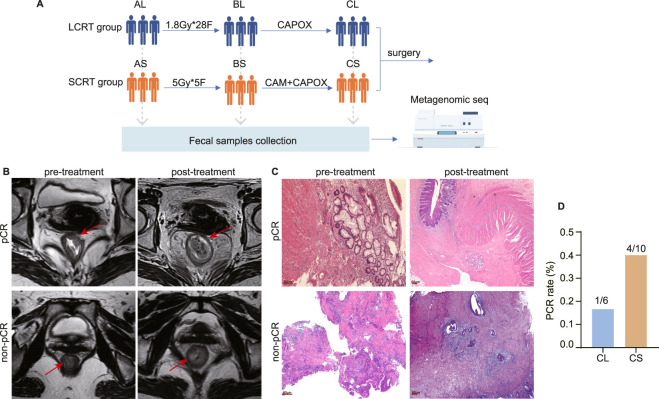
Study design and pathological response outcomes. **(A)** Trial flow chart: patients in the LCRT arm received 1.8 Gy × 28 fractions combined with CAPOX chemotherapy, whereas those in the SCRT arm received 5 Gy × 5 fractions plus CAM + CAPOX. Fecal samples were collected at baseline (AL/AS), post-radiotherapy (BL/BS), and after completion of neoadjuvant treatment (CL/CS) for subsequent metagenomic sequencing. **(B)** Representative baseline and post-treatment MRI scans from pCR and non-pCR patients. **(C)** Representative hematoxylin and eosin pathological (scale bar, 50 μm) images from pCR and non-pCR patients. **(D)** Pathological complete response (pCR) rates in the LCRT and SCRT group.

A total of 36 patients with LARC who participated in the UNION trial between September 2021 and December 2022 were enrolled in this study (n = 17 in the LCRT arm; n = 19 in the SCRT arm) (Figure 1A; Supplementary Figure S1): (1) 3 days before any treatment, with 36 samples obtained (17 LCRT (AL), 19 SCRT (AS)); (2) 3 days after completion of radiotherapy alone, including 12 post-LCRT (BL) and 13 post-SCRT (BS) samples; (3) 6 samples were collected 3 days after completion of LCRT combined with CAPOX (CL) and 10 samples were collected 3 days after completion of SCRT combined with CAM and CAPOX (CS). Key baseline characteristics of all participants are presented in Table 1, patients in LCRT arm and SCRT arm exhibited similar characteristics in terms of gender, age, distance from primary tumor, body mass index (BMI), clinical stage, circumferential resection margin (CRM), extramural venous invasion (EMVI) and neural invasion (all *p* > 0.05).

**TABLE 1:** Patient characteristics.

Characteristics	LCRT group n = 17(%)	SCRT group n = 19(%)	*P*-value
Gender			0.502
Male	12 (70.6)	11 (57.9)	
Female	5 (29.4)	8 (42.1)	
Age (years)	58.7 ± 8.8	57.7 ± 8.5	0.976
Distance from primary tumor to anal verge (cm)	7.38 ± 2.1	6.3 ± 2.4	0.996
BMI (kg/m²)	23.3 ± 2.9	25.3 ± 2.8	0.876
Clinical T category			0.393
cT2	1 (5.9)	0 (0.0)	
cT3	14 (82.4)	18 (94.7)	
cT4	2 (11.8)	1 (5.3)	
Clinical N category			0.399
cN0	2 (11.8)	1 (5.3)	
cN1	11 (64.7)	9 (47.4)	
cN2	4 (23.5)	9 (47.4)	
CRM			0.361
Positive	8 (47.1)	12 (63.2)	
Negative	9 (52.9)	7 (36.8)	
EMVI			0.533
Positive	8 (47.1)	11 (57.9)	
Negative	9 (52.9)	8 (42.1)	
Neural invasion			1.000
Positive	0 (0.0)	1 (5.3)	
Negative	17 (100.0)	18 (94.7)	

Categorical variables were compared using the χ^2^ test or Fisher’s exact test, and continuous variables with the two-sample t-test; *p* < 0.05 was considered statistically significant.

LARC patients received either SCRT or LCRT, followed by two cycles of CAM plus CAPOX or CAPOX alone, followed by radical surgery 1 week later. pCR was defined as the complete absence of viable tumor cells in both the resected primary specimen and all sampled regional lymph nodes (ypT0N0). All sections were independently reviewed by two senior pathologists in a blinded manner. Patients achieving pCR exhibited marked tumor regression with histopathological evidence of complete primary tumor eradication, whereas non-pCR patients demonstrated partial volume reduction yet retained residual viable tumor cells upon post-treatment histological examination (Figures 1B,C). Due to patient compliance and sample-quality control, specimens from 6 patients in the CL group and 10 in the CS group were ultimately available for analysis. And the SCRT group achieved a pCR rate of 40% (4/10), compared with 17% (1/6) in the LCRT group (Figure 1D). These findings are consistent with the early results from our UNION trial, in which the experimental arm achieved a pCR rate of 39.8% (95% CI, 30.7%–49.5%), whereas the control arm reached 15.3% (95% CI, 9.3%–23.0%) (Lin et al., 2024).

### 3.2 Alterations in gut microbiome diversity and structure among LCRT and SCRT group

**FIGURE 2:**
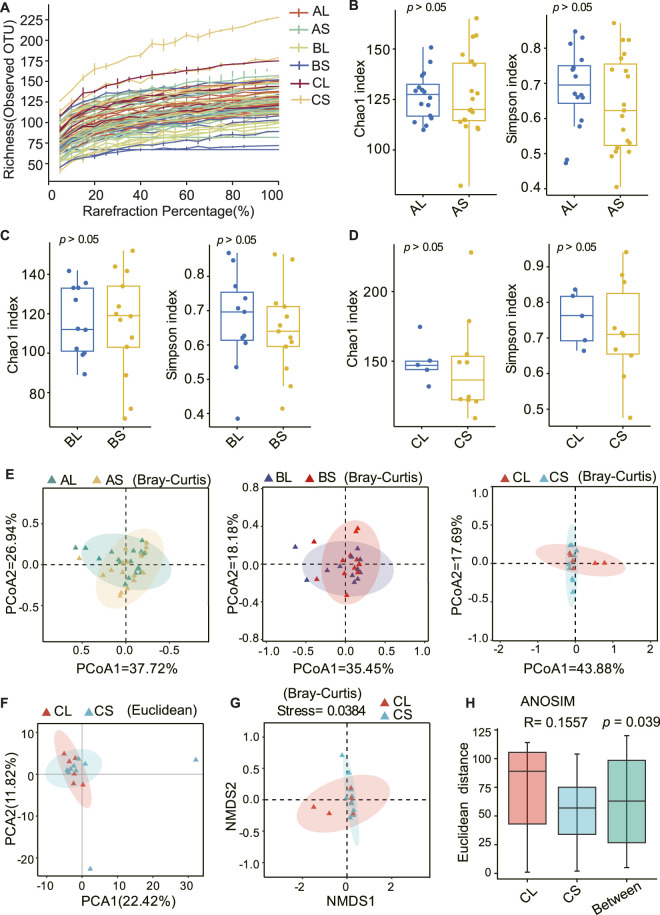
Alterations in gut microbiome diversity and structure among LCRT and SCRT group. **(A)** Rarefaction curves of the metagenomic dataset, the sequencing depth approached saturation of within-sample microbial diversity. **(B–D)** Box plots illustrate the α-diversity of gut microbiomes, as measured by Simpson and Chao 1 index. **(E)** PCoA, **(F)** PCA, and **(G)** NMDS were employed to analyze the gut microbiome composition based on Bray–Curtis (PCoA/NMDS) and Euclidean (PCA) distances. **(H)** ANOSIM were conducted to assess statistically significant differences in β-diversity using Euclidean distance metrics. The box plots display the interquartile range (IQR) with the medians. *p* < 0.05 was considered as statistically significant.

All datasets were quality-filtered with fastp (v0.21.0), and rarefaction curves plateaued for all samples, indicating that sequencing depth had reached saturation and the data comprehensively captured the microbial diversity present (Figure 2A). To depict the characteristics of the intestinal microbiota in two treatment regimens, we conducted a longitudinal comparative analysis of the two groups of patients at multiple treatment time points. Across baseline (AL vs. AS) (Figure 2B), post-radiotherapy (BL vs. BS) (Figure 2C), and post-neoadjuvant-therapy (CL vs. CS) (Figure 2D) time points, no significant differences in α-diversity were observed between the SCRT and LCRT groups (*p* > 0.05). This suggests that, for patients with LARC, the overall richness of the gut microbiota is comparable regardless of whether SCRT followed by CAM plus CAPOX or LCRT followed by CAPOX is employed.

Building on these, we further employed β-diversity analyses to evaluate whether bacterial community structures differed between the two groups at each treatment time point. PCoA of β-diversity based on Bray-Curtis distances at the OTU level showed modest separation between SCRT and LCRT groups after completing neoadjuvant therapy (CS vs. CL) (Figure 2E). However, no significant intergroup differences were observed at baseline or after the radiotherapy. Based on this, we further employed PCA and NMDS to compare the bacterial community structures of the CS group and the CL group. PCA1 and PCA2 explained 22.42% and 11.82% of variance, respectively (Figure 2F). NMDS based on Bray–Curtis distances (stress = 0.0384) showed that the samples of the CL and CS groups were clearly separated in the two-dimensional ranking space, suggesting that there were significant differences in the bacterial community structures of the two groups (Figure 2G). Furthermore, ANOSIM based on Euclidean distance dissimilarity revealed significant differences in β diversity between the two groups (R = 0.1557, *p* = 0.039) (Figure 2H). These findings suggest subtle compositional differences in gut microbiota between treatment arms following neoadjuvant therapy, indicating differential impacts of SCRT and LCRT on microbial community structure. However, the biological significance and clinical relevance of these modest differences remain uncertain given the small sample size and limited effect magnitude, and require validation in larger, adequately powered cohorts.

### 3.3 Distinct gut microbiome between the LCRT and SCRT groups after completion of neoadjuvant treatment

**FIGURE 3:**
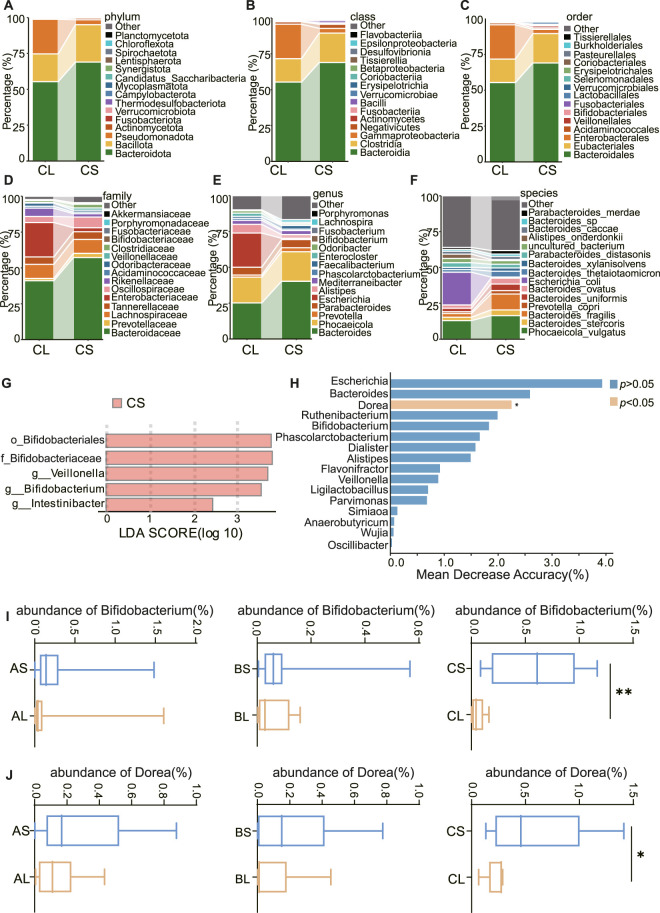
Distinct fecal microbiota between the LCRT and SCRT groups after completion of neoadjuvant treatment. The stacked bar chart shows the 15 most abundant taxa at the phylum **(A)**, class **(B)**, order **(C)**, family **(D)**, genus **(E)**, and species **(F)** levels in the two groups. **(G)** LEfSe analysis (LDA score > 2.0) identified to find significantly differing gut microbial between the two groups, and the two-tailed test analysis was used to find the different species between the two groups. **(H)** The random-forest model identified the bacterial genera that best differentiated the two groups. The histogram showed the relative abundance of *Bifidobacterium* **(I)** and *Dorea* **(J)** in the two groups. The box plots display the interquartile range (IQR) with the medians. *p < 0.05, **p < 0.01.

The preliminary results indicated that the efficacy differences among various neoadjuvant treatment modalities were accompanied by significant changes in the intestinal microbiota. Based on these findings, we compared post-treatment microbial profiles for patients receiving LCRT plus CAPOX versus SCRT plus CAM and CAPOX (CL vs. CS). The top 15 taxa at the phylum, class, order, family, genus, and species levels were identified for each group. Compared with the CL group, the CS group exhibited markedly higher relative abundance of the *Bacteroidetes* phylum and its subordinate groups across all taxonomic levels from phylum to genus (Figures 3A–E), alongside enrichment of beneficial symbiotic bacteria including *Faecalibacterium* and *Bifidobacterium* (Figures 3E,F). Notably, species-level analysis revealed distinct compositional differences within the *Bacteroides* genus: the CS group displayed a more diverse assemblage enriched with beneficial members such as *B. uniformis*, *Bacteroides ovatus*, and *B. thetaiotaomicron* (Figure 3F), which have been associated with anti-inflammatory properties, mucosal homeostasis maintenance, and metabolic health benefits (Hayase et al., 2024; Wang et al., 2024; Weis et al., 2025). *Bacteroides fragilis* was also more abundant in the CS group. While certain strains of this species are enterotoxigenic and associated with carcinogenesis (Dejea et al., 2018), it is noteworthy that non-enterotoxigenic *B. fragilis* (NTBF) represents a prevalent commensal strain that contributes to mucosal immune homeostasis (Yang et al., 2025). Given the favorable treatment response observed in the CS group, the expansion of *B. fragilis* likely reflects enrichment of beneficial NTBF variants rather than toxigenic strains.

To further characterize the microbiota signatures distinguishing the CL and CS groups, we conducted LEfSe analysis (LDA score > 2, *p* < 0.05), which revealed significant enrichment of *o_Bifidobacteriales*, *f_Bifidobacteriaceae*, *g_Veillonella*, *g_Bifidobacterium* and *g_Intestinibacter* (Figure 3G) in the gut microbiota of CS patients. Random-forest analysis (Figure 3H) further revealed that the genera *Escherichia*, *Bacteroides*, *Dorea*, *Ruthenibacterium*, *Bifidobacterium*, *Phascolarctobacterium*, and *Dialister* were enriched in the CS group, among which the enrichment of *Dorea* was statistically significant (*p* < 0.05). Assessment of variable importance identified *Escherichia* (mean decrease in Gini = 0.50), *Bifidobacterium* (0.40), *Bacteroides* (0.21), and *Dorea* (0.19) as the top discriminatory taxa between groups. Integrating differential findings, we next tracked the relative abundances of these key taxa across treatment time points. The results showed that at baseline (AL vs. AS) and post-radiotherapy (BL vs. BS), no significant differences in these taxa were observed. However, after the completion of the entire treatment regimen, the CS group demonstrated significantly higher levels of *Bifidobacterium* and *Dorea* than the CL group (Figures 3I,J). These results indicate that the intestinal microbiota exhibit significant heterogeneity under different treatment regimens. It is noteworthy that the *Bifidobacterium* and *Dorea* that were significantly enriched in the CS group have both been reported to possess potential immunomodulatory functions (Matson et al., 2018; Juárez-Fernández et al., 2023).

### 3.4 Association of the microbial communities with outcomes in SCRT arm

Accumulating preclinical and clinical data now firmly establish that the gut microbiota modulates anti-tumor immunity and shapes the response to immunotherapies (Lu et al., 2022; Li et al., 2024). Our previous results also showed that, compared with LCRT combined with chemotherapy, the abundances of *Bifidobacterium* and *Dorea* significantly increased after SCR combined with chemotherapy and camrelizumab. We next performed a longitudinal analysis to delineate and compare the dynamic trajectories of *Bifidobacterium* and *Dorea* under the two therapeutic regimens.

**FIGURE 4:**
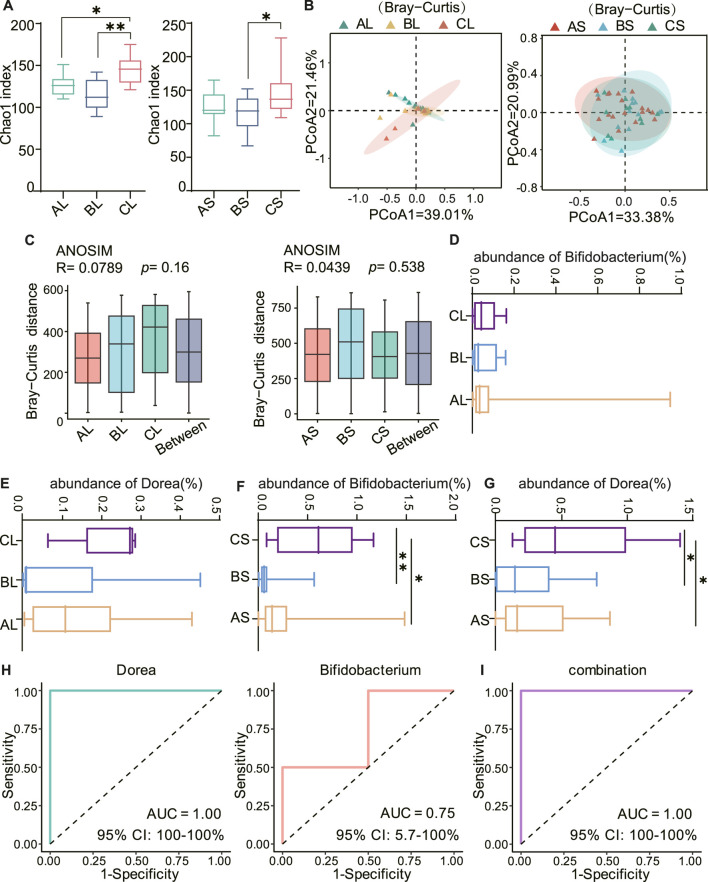
Association of the microbial communities with outcomes in SCRT arm. **(A)** Box plots illustrate the α-diversity (Chao1 index) of gut microbiomes in the LCRT and SCRT groups at baseline, post-radiotherapy, and after completion of neoadjuvant treatment. **(B)** PCoA was employed to analyze the gut microbiome composition based on Bray–Curtis distance. **(C)** ANOSIM were conducted to assess statistically significant differences in β-diversity using Bray-Curtis distance. The histogram showed the relative abundance of *Bifidobacterium* **(D)** and *Dorea* **(E)** in the LCRT group at baseline, post-radiotherapy, and after completion of neoadjuvant treatment. The histogram showed the relative abundance of *Bifidobacterium* **(F)** and *Dorea* **(G)** in the SCRT group at baseline, post-radiotherapy, and after completion of neoadjuvant treatment. **(H)** ROC curves for *Dorea* (AUC = 1.00, 95% CI: 100%–100%) and *Bifidobacterium* (AUC = 0.75, 95% CI: 5.7%–100%) in predicting treatment response (pCR vs. non-pCR) following SCRT combined with camrelizumab and chemotherapy. **(I)** ROC curve for the combination (AUC = 1.00, 95% CI: 100%–100%). Note the wide CI for *Bifidobacterium* due to small sample size (n = 10). These findings are exploratory and require independent validation. The box plots display the interquartile range (IQR) with the medians. *p < 0.05, **p < 0.01.

We first assessed α-diversity at multiple time points under both regimens. Compared with baseline, both treatments significantly increased microbial diversity, as reflected by markedly elevated Chao1 indices (Figure 4A). β-diversity analyses were further employed to compare bacterial community structures of the two schemes at different time points. PCoA revealed no discernible separation between the pre-treatment and post-treatment samples within either group (Figure 4B). ANOSIM based on Bray-Curtis distance further confirmed that neither group exhibited a statistically significant shift in β-diversity before versus after treatment (Figure 4C). These analyses demonstrate that both interventions significantly enriched microbial species richness, as evidenced by significant increases in α-diversity, yet elicited no discernible shifts in overall community structure (β-diversity). Nevertheless, given that we had found that the relative abundances of *Bifidobacterium* and *Dorea* were specifically enriched in SCRT group compared with LCRT group, we next focused on these two taxa and compared their differential abundance before versus after treatment under both regimens. The results showed that in the LCRT group, the relative abundances of *Bifidobacterium* and *Dorea* did not change significantly before versus after treatment (Figures 4D,E). Strikingly, both taxa exhibited a marked increase in relative abundance after SCRT followed by CAM plus CAPOX (Figures 4F,G). These two taxa recognized for their immunomodulatory potential, imply that they may contribute to the response observed with SCRT followed by chemotherapy and immunotherapy.

Studies have shown that *Bifidobacterium* and *Dorea* potentiate anti-tumor immunity and synergistically improve the efficacy of anti-programmed death-ligand 1 (PD-L1) therapy, and both genera are enriched in responders to neoadjuvant therapy for LARC (Ayelet et al., 2015; Yi et al., 2021). In view of these findings, we next constructed an ROC-based predictive model using the relative abundances of these two genera as core variables to assess their value in forecasting the response to SCRT combined with camrelizumab and chemotherapy. *Dorea* alone achieved an AUC of 1.00, while *Bifidobacterium* alone showed an AUC of 0.75 (Figure 4H). The extremely wide CI for *Bifidobacterium* reflects high uncertainty due to small sample size (n = 10). The combined model yielded an AUC of 1.0 (Figure 4I). Notably, the exceptionally wide CI for *Bifidobacterium* underscores the high uncertainty stemming from the limited sample size (n = 10). These near-perfect AUC values likely indicate model overfitting; consequently, the true predictive performance in independent cohorts is expected to be substantially lower. As an exploratory finding, this signature requires further validation in future studies before clinical consideration.

### 3.5 Functional annotation analysis of gut microbiomes with KEGG and CAZy

**FIGURE 5:**
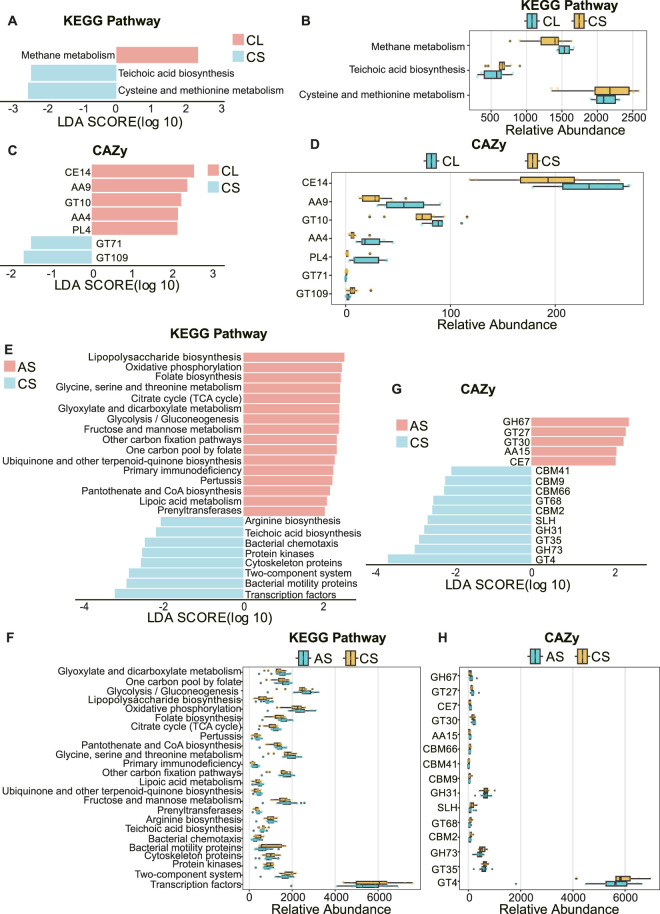
Functional annotation analysis of gut microbiomes with KEGG and CAZy. **(A)** According to KEGG, python was used to sort out the content table of each sample gene in different metabolic pathways, then employed LEfSe to identify differential pathways between CL and CS. **(B)** Enriched KEGG ortholog markers within the differential pathways were compared between CL and CS. **(C)** CAZy profiles were compared between CL and CS. **(D)** Functional comparisons based on CAZy classification were conducted between CL and CS populations. **(E)** LEfSe was employed to identify differential pathways between AS and CS based on KEGG. **(F)** KEGG ortholog markers enriched in differentially abundant metabolic pathways were compared between the two cohorts. **(G)** CAZy comparisons were conducted between AS and CS. **(H)** Functional comparisons based on CAZy classification were conducted between the entire populations of the AS and CS.

LEfSe (LDA > 2) was applied to identify differentially enriched KEGG pathways and CAZy families between the CS and CL groups. Methionine metabolism was significantly enriched in the CL group, whereas teichoic acid biosynthesis and cysteine and methionine metabolism was significantly enriched in the CS group (Figures 5A,B). For carbohydrate metabolism, the CS group exhibited enrichment in glycosyl transferases (GT71, GT109), whereas the CL group displayed higher abundances of carbohydrate esterases (CE14) (Figures 5C,D).

Likewise, the same analytical pipeline was applied to examine the functional shifts in the gut microbiome of the SCRT group between baseline (AS) and post-treatment (CS). At baseline, the gut microbiome was predominantly characterized by pro-inflammatory and energy-sustaining pathways, such as lipopolysaccharide biosynthesis, oxidative phosphorylation, and folate biosynthesis. After treatment, the functional profile shifted markedly toward regulatory and motility-associated pathways (transcription factors, teichoic acid biosynthesis, and arginine biosynthesis) (Figures 5E,F; Supplementary Table S1), indicating a transition from an inflammatory-stress state to a metabolism-adaptation state (Mills et al., 2016; Qian et al., 2024). With respect to carbohydrate metabolism, baseline samples were dominated by glycoside hydrolases and carbohydrate esterases, whereas post-treatment microbiota exhibited marked increases in carbohydrate-binding modules (CBMs 41, 9, 66, 2) and glycosyl transferases (Figures 5G,H; Supplementary Table S2), indicating enhanced capacities for carbohydrate recognition and synthesis.

## 4 Discussion

A defining characteristic of colorectal cancer is its intimate interplay with the gut microbiota, which serves as a pivotal constituent of the tumor microenvironment. Targeted modulation of the gut microbiome opens novel prospects for the chemical prevention, non-invasive screening and precise treatment of colorectal cancer (Liu Y. et al., 2023; Wong and Yu, 2023). Emerging evidence reveals that LARC patients exhibit distinct gut microbial profiles according to their response to neoadjuvant chemoradiotherapy, and these microbial signatures can be used to predict the therapeutic efficacy (Qu et al., 2023; Teng et al., 2023). To our knowledge, this study is the first to profile and compare gut microbiome alterations in LARC patients treated with either SCRT followed by CAM and CAPOX or LCRT combined with CAPOX. We preliminarily identified differentially abundant taxa and explored a microbiome-based classifier associated with response to SCRT with camrelizumab and chemotherapy, providing candidate signatures for future validation in support of individualized treatment decisions.

Prior studies have demonstrated that the heterogeneous response of LARC patients to neoadjuvant therapy is closely associated with gut microbiota composition, and microbial signatures can reliably predict treatment efficacy (Sun et al., 2023; White et al., 2023). The UNION study conducted in our center showed that, neoadjuvant SCRT followed by CAM and CAPOX achieved a significantly higher pCR rate than LCRT followed by CAPOX in LARC patients. In the present study, we further observed that the post-treatment gut microbiota structure differed significantly between patients in SCRT arm and those in LCRT arm. Notably, we observed a significant enrichment of *Bifidobacterium* and *Dorea* in the SCRT group relative to the LCRT group. Furthermore, longitudinal analysis showed that the relative abundances of *Bifidobacterium* and *Dorea* showed significant changes before versus after the treatment only in the SCRT group. Compared with the levels at the baseline and after the end of short-course radiotherapy, the abundances of these two bacteria significantly increased during the subsequent CAM + CAPOX treatment. Conversely, in the LCRT group, the relative abundances of *Bifidobacterium* and *Dorea* remained stable across all observation time points without significant fluctuations. These findings indicate that the efficacy disparity between the two regimens may be driven by microbiota changes specifically induced during the chemo-immunotherapy period, and may be closely related to the immune response elicited by CAM (Pan et al., 2024).

This temporal pattern aligns with prior evidence associating *Bifidobacterium* with favorable prognosis in colorectal cancer. Additionally, our observation that *Dorea* enrichment correlates with response is consistent with the hypothesis that this genus may serve as a candidate biomarker for predicting nCRT responses in LARC patients, pending validation in larger cohorts (Yi et al., 2021; Ugai et al., 2025). Previous longitudinal studies tracking gut microbiota throughout nCRT in LARC patients have linked dynamic trajectories of taxa such as *Bacteroides vulgatus* to treatment resistance (Teng et al., 2023; Wong et al., 2023), underscoring the superiority of longitudinal monitoring over single-timepoint assessment. However, the translational potential of leveraging these temporal patterns for predictive modeling remains underexplored. Integrating longitudinal microbiome monitoring (e.g., serial sampling before, during, and after treatment) with dynamic modeling represents a promising strategy to enhance personalized treatment prediction in LARC. Nevertheless, existing studies have not directly validated the hypothesis that temporal dynamic features significantly outperform single-timepoint models. Furthermore, the generalizability of our findings remains tentative due to the limited sample size of the current study, necessitating future validation through large-scale prospective cohort studies. The potential mechanisms underlying the observed associations warrant consideration. Previous experimental studies have demonstrated that *Bifidobacterium pseudolongum* promotes the differentiation of CD8^+^ T cells into memory phenotype by secreting L-arginine, and enhances the therapeutic effect of anti-CTLA-4 in colorectal cancer (Nan et al., 2025). *Bifidobacterium* isolated from immune checkpoint blockade (ICB)-treated colorectal cancers functions as a key commensal intestinal species that enhances ICB efficacy in mouse models of intestinal and epithelial tumors by amplifying a cDC-dependent TH1 cell circuit (Mager et al., 2020). Moreover, *Bifidobacterium* can promote the generation of key metabolites like butyric acid in the intestinal cavity, and significantly accumulate α-ketoglutaric acid, N-acetyl-L-glutamic acid, and pyridoxine from the blood. These metabolic signals work together to reshape the TME by promoting cytotoxic T-lymphocyte infiltration and activation while concurrently suppressing regulatory T cell function, thereby substantially improving the efficacy of immunotherapy (Gao et al., 2023). Our observation of *Bifidobacterium* enrichment in responders aligns with these reported mechanisms, although causality remains to be established given the observational nature of this dataset. Whereas *Bifidobacterium* has been extensively characterized for its contribution to immunotherapeutic efficacy, *Dorea* remains under-studied. Nevertheless, accumulating evidence now implicates this genus is also implicated in cancer immunotherapeutic outcomes. The genomic structural variations of *Dorea* are significantly correlated with the clinical benefits of immune checkpoint inhibitor (ICI), these SV regions are enriched for genes involved in glucose metabolism and may modulate host metabolic-immune axis, thereby influencing ICI efficacy and prognosis (Liu R. et al., 2023). Our observation that *Dorea* abundance is associated with response in the SCRT cohort lends support to the hypothesis of its immunomodulatory contribution. Future experimental studies are needed to test whether *Dorea* plays a causal role in this treatment context.

In addition, the metabolites derived from the gut microbiome play a crucial role in cancer progression and treatment (Chen et al., 2022; Coker et al., 2022). In this study, we also observed that the arginine metabolism pathway was significantly enriched in patients received the treatment of SCRT followed by CAM and CAPOX. Arginine is a multifunctional amino acid whose biosynthesis is carried out not only by host cells but also actively by the gut commensal *Bifidobacterium*. As the core precursor of multiple metabolic pathways, arginine derived from *Bifidobacterium* can regulate cell proliferation and significantly reshape the host’s immune function (Canale et al., 2021; Nüse et al., 2023). Moreover, L-arginine interacts with transcriptional regulators to enhance glycolysis and mitochondrial activity, thereby prolonging T cell persistence; it also promotes the generation of central memory T cells (TCM) and markedly enhances antitumor efficacy (Geiger et al., 2016). Another metabolite of *Bifidobacterium*, butyric acid, can directly downregulate PD-L1 and IL-10 in peripheral immune cells and tumor-infiltrating lymphocytes, thereby reversing the immunosuppressive microenvironment and inhibiting tumor growth (Lee et al., 2024). Butyrate binds to Toll-like receptor 5 (TLR5) on CD8^+^ T cells, activates nuclear factor-κB (NF-κB) signalling, and induces the expression of granzyme B (GZMB), interferon-γ (IFN-γ) and tumour necrosis factor-α (TNF-α) in CD8^+^ T cells, thereby inhibiting tumour growth and enhancing anti-programmed death receptor 1 (PD-1) therapeutic responses in tumour-bearing mice (Kang et al., 2023). Interestingly, *Dorea*, which was significantly enriched in the SCRT group, can secrete acetic and lactic acids (Palmnäs-Bédard et al., 2022). These two short-chain fatty acids can be further metabolized by the gut microbiota into butyric acid, thereby indirectly enhancing the immune regulatory effect mediated by butyric acid (Louis and Flint, 2017). Whether *Dorea* directly participates in the immune regulation of the TME still requires experimental verification.

At baseline, the gut microbiome exhibited a functional landscape characterized by lipopolysaccharide biosynthesis, oxidative phosphorylation, and folate metabolism, reflecting a pro-inflammatory state. After treatment, a significant shift was observed, with pronounced enrichment in transcription factors, two-component systems, bacterial chemotaxis, cytoskeletal proteins, and protein kinases, reflecting an enhanced capacity for signal transduction, and gene regulation. These results indicate a functional transition from an inflammation-associated stress condition to a state geared toward metabolic adaptation and homeostatic regulation. This functional transformation was also confirmed on the CAZymes spectrum. Prior to treatment, the enriched enzymes (such as GH67, GT27, and CE7) usually participate in the decomposition or modification of specific oligosaccharides, with relatively simple functions and a bias towards catabolic metabolism (Janeček et al., 2014; Alexander and Locher, 2023). After treatment, the carbohydrate metabolic capacity of the microbial community became more diverse and complex. The most notable change was a substantial increase in the diversity and abundance of carbohydrate-binding modules (CBMs, including CBM41, CBM9, CBM66, and CBM2) (You et al., 2024).

This study has three limitations. Firstly, this represents an exploratory pilot analysis with a modest sample size from a single center, which may limit the generalizability of our findings. Participants’ similar geographical backgrounds, dietary patterns, and lifestyle habits further constrain applicability to broader populations. Validation within the ongoing UNION phase III multicenter trial, currently expanding to additional centers, will address this limitation and enhance the reliability of microbiome-based predictive models. Secondly, the limited number of pCR events and incomplete longitudinal sampling constrained our ability to perform robust multivariable analyses simultaneously adjusting for multiple clinical covariates (e.g., age, sex, cT stage, and baseline CEA alongside microbiome features). According to the Events Per Variable (EPV) principle, inclusion of numerous covariates with limited pCR events would risk model overfitting and unstable estimates. Finally, this study focused exclusively on fecal samples and did not incorporate other microbial sources (e.g., tumor tissue or saliva). Therefore, the results may not fully capture the overall composition and functional characteristics of the human microbiota.

## 5 Conclusion

In summary, this exploratory study proposes that the response of LARC patients to SCRT combined with CAM plus CAPOX is associated with features of the gut microbiome. We preliminarily identified two microbial markers related to the therapeutic effect and explored potential mechanisms by which these microorganisms may affect the treatment outcomes. Distinct shifts in the gut microbiota were observed between the SCRT and LCRT cohorts. Specifically, *Bifidobacterium* and *Dorea* were significantly enriched following completion of SCRT sequential CAM and CAPOX therapy, and showed potential to distinguish between responders and non-responders in the SCRT group. Nevertheless, given the limited sample size, these findings are primarily hypothesis-generating and necessitate validation in larger, independent cohorts prior to clinical implementation. Future strategies based on probiotics or microbiome intervention warrant further investigation as potential treatment directions.

## Data Availability

The datasets presented in this study can be found in online repositories. The names of the repository/repositories and accession number(s) can be found below: https://www.cncb.ac.cn/, PRJCA058322.
