# Supplementary material for: Gut microbiome components predict response to neoadjuvant short-course radiotherapy followed by camrelizumab and chemotherapy in locally advanced rectal cancer (UNION): a prospective study
Source: Front Pharmacol. 2026 May 29;17:1829108. doi: 10.3389/fphar.2026.1829108 (PMC13260075; doi:10.3389/fphar.2026.1829108)
Supplement: Supplementary file 1 [file Supplementaryfile1.docx]

**Supplementary Figure S1. Cohort description and sample flow**


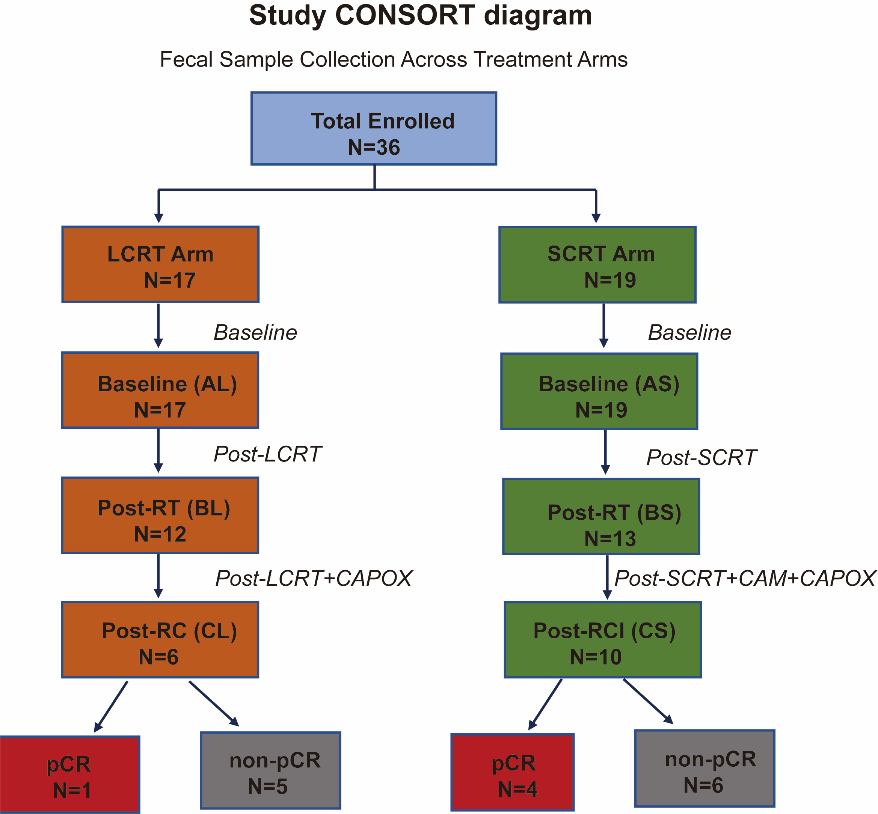


**Figure S1. CONSORT diagram of patient grouping, fecal sample collection, and treatment outcomes.** 36 patients were randomized to long-course radiotherapy (LCRT, n=17) or short-course radiotherapy (SCRT, n=19) arms. Fecal samples were collected at three time points: baseline (AL/AS), post-radiotherapy (BL/BS), and post-neoadjuvant chemotherapy (CL/CS). Pathological complete response (pCR) status was assessed as the primary treatment outcome. The diagram illustrates the substantial attrition at each collection time point, with only 16 patients (6 in LCRT arm and 10 in SCRT arm) completing all three sampling time points. The small sample sizes at the final time point, particularly the limited pCR cases (1 in LCRT, 4 in SCRT).
