# Supplementary material for: Gut microbiome components predict response to neoadjuvant short-course radiotherapy followed by camrelizumab and chemotherapy in locally advanced rectal cancer (UNION): a prospective study
Source: Front Pharmacol. 2026 May 29;17:1829108. doi: 10.3389/fphar.2026.1829108 (PMC13260075; doi:10.3389/fphar.2026.1829108)
Supplement: Supplementary file 2 [file Table1.docx]

**Supplementary Tables S1. Results of LEfSe analysis on KEGG pathways**

| **Pathway** | **Enriched group** | **LDA score** | ***P*-value** | **q-value** |
| --- | --- | --- | --- | --- |
| Lipopolysaccharide biosynthesis | AS | 2.521776497 | 0.031044028* | 0.046406104^#^ |
| Oxidative phosphorylation | AS | 2.457335089 | 0.024558711* | 0.046406104^#^ |
| Folate biosynthesis | AS | 2.432037768 | 0.034804578* | 0.046406104^#^ |
| Glycine, serine and threonine metabolism | AS | 2.414039722 | 0.038947456* | 0.046736947^#^ |
| Citrate cycle (TCA cycle) | AS | 2.410578138 | 0.024558711* | 0.046406104^#^ |
| Glyoxylate and dicarboxylate metabolism | AS | 2.394836116 | 0.011616891* | 0.046406104^#^ |
| Glycolysis / Gluconeogenesis | AS | 2.394515383 | 0.021781463* | 0.046406104^#^ |
| Fructose and mannose metabolism | AS | 2.379895046 | 0.048498375* | 0.048498375^#^ |
| Other carbon fixation pathways | AS | 2.329690256 | 0.024558711* | 0.046406104^#^ |
| One carbon pool by folate | AS | 2.329681775 | 0.017036288* | 0.046406104^#^ |
| Ubiquinone and other terpenoid-quinone biosynthesis | AS | 2.281643815 | 0.031044028* | 0.046406104^#^ |
| Primary immunodeficiency | AS | 2.24587859 | 0.038947456* | 0.046736947^#^ |
| Pertussis | AS | 2.220264635 | 0.034804578* | 0.046406104^#^ |
| Pantothenate and CoA biosynthesis | AS | 2.161197249 | 0.010185826* | 0.046406104^#^ |
| Lipoic acid metabolism | AS | 2.085537891 | 0.048498375* | 0.048498375^#^ |
| Prenyltransferases | AS | 2.031527794 | 0.048498375* | 0.048498375^#^ |
| Arginine biosynthesis | CS | 2.065708149 | 0.048498375* | 0.048498375^#^ |
| Teichoic acid biosynthesis | CS | 2.18363085 | 0.004444558** | 0.046406104^#^ |
| Bacterial motility proteins | CS | 2.919857365 | 0.027637698* | 0.046406104^#^ |
| Protein kinases | CS | 2.5301487 | 0.013223601* | 0.046406104^#^ |
| Cytoskeleton proteins | CS | 2.563483772 | 0.034804578* | 0.046406104^#^ |
| Two-component system | CS | 2.860773905 | 0.007785749** | 0.046406104^#^ |
| Bacterial chemotaxis | CS | 2.463512041 | 0.027637698* | 0.046406104^#^ |
| Transcription factors | CS | 3.214622431 | 0.010185826* | 0.046406104^#^ |

The q-value was determined with Benjamini-Hochberg method. **P* < 0.05, ***P* < 0.01; ^#^q < 0.05.
