# Supplementary material for: Gut microbiome components predict response to neoadjuvant short-course radiotherapy followed by camrelizumab and chemotherapy in locally advanced rectal cancer (UNION): a prospective study
Source: Front Pharmacol. 2026 May 29;17:1829108. doi: 10.3389/fphar.2026.1829108 (PMC13260075; doi:10.3389/fphar.2026.1829108)
Supplement: Supplementary file 3 [file Table2.docx]

**Supplementary Tables S2. Results of LEfSe analysis on CAZy** **families**

| **CAZy family** | **Enriched group** | **LDA-value** | ***P*-value** | **q-value** |
| --- | --- | --- | --- | --- |
| GH67 | AS | 2.498923323 | 0.034804578* | 0.040159128^#^ |
| GT27 | AS | 2.421774123 | 0.013223601* | 0.032193686^#^ |
| GT30 | AS | 2.365949855 | 0.027637698* | 0.040159128^#^ |
| AA15 | AS | 2.182689835 | 0.01928165* | 0.036153094^#^ |
| CE7 | AS | 2.164436687 | 0.043501933* | 0.043501933^#^ |
| CBM41 | CS | 2.050905143 | 0.000345063*** | 0.005175945^##^ |
| CBM9 | CS | 2.206389646 | 0.013223601* | 0.032193686^#^ |
| CBM66 | CS | 2.234613014 | 0.027637698* | 0.040159128^#^ |
| GT68 | CS | 2.512362239 | 0.004444558** | 0.032193686^#^ |
| CBM2 | CS | 2.540410078 | 0.010185826* | 0.032193686^#^ |
| SLH | CS | 2.659670695 | 0.01502372* | 0.032193686^#^ |
| GH31 | CS | 2.745100346 | 0.043501933* | 0.043501933^#^ |
| GT35 | CS | 2.879829386 | 0.034804578* | 0.040159128^#^ |
| GH73 | CS | 2.989512539 | 0.013223601* | 0.032193686^#^ |
| GT4 | CS | 3.679552917 | 0.034804578* | 0.040159128^#^ |

The q-value was determined with Benjamini-Hochberg method. **P* < 0.05, ***P* < 0.01, ****P* < 0.001; ^#^q < 0.05, ^##^q < 0.01.
