# Supplementary material for: Gut microbiome components predict response to neoadjuvant short-course radiotherapy followed by camrelizumab and chemotherapy in locally advanced rectal cancer (UNION): a prospective study
Source: Front Pharmacol. 2026 May 29;17:1829108. doi: 10.3389/fphar.2026.1829108 (PMC13260075; doi:10.3389/fphar.2026.1829108)
Supplement: Supplementary file 4 [file Table3.docx]

**Supplementary Tables S3. Group Assignment and Sample IDs for Sequencing**

|  | non-pCR | pCR |
| --- | --- | --- |
| AL | A1, A2, A3, A4, A5, A9, A11, A13, A18, A19, A20 | A7, A8, A10, A15, A16, A17 |
| BL | B1, B2, B5, B8, B9, B10, Met1, Met19, Met25 | B6, B7, Met31 |
| CL | C2, C3, Met4, Met9, Met20 | Met5 |
| AS | A23, A24, A25, A26, A28, A31, A32, A33, A34, Met6, Met21, Met12 | A21, A22, A27, A30, A35, A37, Met28 |
| BS | B11, B13, B14, B15, B16, B20, Met7, Met23 | B12, B17, B19, Met17, Met11 |
| CS | C7, C8, C9, C11, Met15, Met22, | C12, Met3, Met27, Met30 |
